# Assessment and Certification of Neonatal Incubator Sensors through an Inferential Neural Network

**DOI:** 10.3390/s131115613

**Published:** 2013-11-15

**Authors:** José Medeiros de Araújo Júnior, José Maria Pires de Menezes Júnior, Alberto Alexandre Moura de Albuquerque, Otacílio da Mota Almeida, Fábio Meneghetti Ugulino de Araújo

**Affiliations:** 1 Electrical Engineering Course, Federal University of Piauí (UFPI), 64049-550, Teresina, Piauí, Brazil; E-Mails: josemenezesjr@ufpi.edu.br (J.M.P.M.J.); otacilio@ufpi.edu.br (O.M.A.); 2 Department of Electrical Engineering, Federal University of Ceará (UFC), 60020-181, Fortaleza, Ceará, Brazil; E-Mail: alberto.alexandre@gmail.com; 3 Department of Computer Engineering and Automation, Federal University of Rio Grande do Norte (UFRN), 59078-900, Natal, Rio Grande do Norte, Brazil; E-Mail: meneghet@dca.ufrn.br

**Keywords:** sensors, inferential neural network, nonlinear identification, neonatal incubator, certification procedure

## Abstract

Measurement and diagnostic systems based on electronic sensors have been increasingly essential in the standardization of hospital equipment. The technical standard IEC (International Electrotechnical Commission) 60601-2-19 establishes requirements for neonatal incubators and specifies the calibration procedure and validation tests for such devices using sensors systems. This paper proposes a new procedure based on an inferential neural network to evaluate and calibrate a neonatal incubator. The proposal presents significant advantages over the standard calibration process, *i.e.*, the number of sensors is drastically reduced, and it runs with the incubator under operation. Since the sensors used in the new calibration process are already installed in the commercial incubator, no additional hardware is necessary; and the calibration necessity can be diagnosed in real time without the presence of technical professionals in the neonatal intensive care unit (NICU). Experimental tests involving the aforementioned calibration system are carried out in a commercial incubator in order to validate the proposal.

## 1. Introduction

Newborns with health complications and premature birth have great difficulty in regulating their body temperature because of various reasons, such as a high metabolism rate caused by the conditions of an illness, low birth weight and a high rate of surface to body volume, which causes a high amount of energy per kilogram to be lost when compared to an adult. Within this context, a neonatal incubator helps in taking care of the health of newborns [1]. A neonatal incubator must be seen as a thermoneutral environment, which provides favorable conditions that assure the minimum energy expenditure of the newborn while body temperature is within a safe range [2].

A neonatal incubator is one of the most important pieces of neonatal intensive care unit (NICU) equipment [3,4]. For every 1,000 Brazilian children born in 2010, about 8.7 individuals died in the first week of life, while 2.6 individuals died between the seventh and 27th day of life, according to the Brazilian Ministry of Health [5]. Within this period of early life, the incubators are fundamental tools for reducing the risk of mortality and diseases. The incubator provides an adequate microclimate, so that newborn infants can overcome the first occurrences of diseases, and it also controls skin temperature and the relative humidity [6].

As with any other electro-medical equipment, a neonatal incubator must be calibrated periodically, because its malfunction may cause serious damage to the newborn's health or even lead to the newborn's death. The technical standard IEC 60601-2-19 establishes operating specifications for neonatal incubators, so that a safe environment can be offered for newborns [7]. Such specifications are verified by performing several tests, including the application of input signals to the temperature, humidity and air flow actuators, with the aim of analyzing the behavior of the aforementioned variables at specific points inside the incubator.

In order to perform the aforementioned tests, the incubator is removed from service, and a calibration system is installed, so that temperature and humidity data are provided for a standardized calibration procedure. Considering that it is necessary to turn off the incubator to run the tests and, also, that the number of available incubators in neonatal centers is limited in most Brazilian hospitals, a maintenance strategy that minimizes the incubator down time will prove to be very useful.

In this work, a literature survey is carried out by considering the published studies addressing the identification and control of the dynamics involving temperature and humidity in neonatal incubators. Zermani *et al.* [8] use genetic algorithms (GA) to estimate the parameters of NARMA (nonlinear auto-regressive moving average) and ARX (autoregressive model with external input) models, which identify the dynamic properties of humidity in the newborn incubator. Furthermore, a comparative study was made between a proportional integral derivative (PID) controller and a model-based predictive control (MPC), where the parameters of the PID controller and the cost function of MPC were optimized using GA [8]. Such controllers are used to adjust the humidity inside the incubator. The simulated results presented by the researchers have demonstrated that the MPC controller has a better performance when compared with the PID one.

Zermani *et al.* [9] design an indirect adaptive generalized predictive controller (IAGPC) for the temperature loop in a newborn incubator. In this case, the process is identified by an ARX structure, whose parameters are updated in real time to fit the process changes. The authors establish a comparison among three controllers: (i) ON-OFF; (ii) PID and (iii) IAGPC. Experimental results show that IAGPC is the most efficient controller. In Neto *et al.* [10], the researchers proposed the application of multivariable PI control strategies for the humidity and temperature control loops in the neonatal incubator. The evaluated TITO (two input two output) system was decoupled into four independent loops. In order to ensure the robustness and stability of the whole control system, PI tuning-based methods, such as modified Ziegler-Nichols and revised BLT (Biggest Log-Module Tuning) were then developed.

Amer and Al-Aubidy [11] declared that it is essential to detect any abnormal condition in the premature birth incubator system as soon as possible. In their work, a novel technique using an artificial neural network (ANN) is used in order to simulate adequate incubator control for taking care of premature births. The outputs of the sensors that indicate the temperature, humidity and oxygen concentration of the incubator internal environment are the inputs of an ANN, which identifies the corresponding case and decides the suitable reaction based upon previous training. According to the authors, the ANNs have been consolidated as a powerful tool to be used in the control and identification of dynamic systems.

ANNs are massively-distributed parallel structures, consisting of simple processing units known as neurons, which have a natural propensity for storing experimental knowledge and making it available for use [12]. Among the ANN properties, some of the most relevant ones are the intrinsic nonlinearity, the generalization capabilities and adaptability, fault tolerance and the universal approximation capability [13].

In the literature, several studies report applications of ANNs as a potential technique for identification of the dynamical behavior for temperature and humidity inside controlled environments. Although these next briefly described works are not directly related to neonatal incubators, there is some similarity with the scope of this paper. Martnez *et al.* [14] presented a system based on an ANN for the estimation and prediction of environmental variables related to the tobacco drying process. This system was validated with temperature and relative humidity data obtained from a real tobacco dryer through a wireless sensor network (WSN).

In another paper, Ferreira *et al.* [15] developed an intelligent, light-weight and portable sensor, using ANN models as a time-series predictor system in order to obtain accurate measurements for global solar radiation and atmospheric temperature. This sensor can be applied in several areas, such as in agriculture, renewable energy and energy management or thermal comfort in buildings. ANNs were also applied by Salazar *et al.* [16] in order to predict the temperature and relative humidity inside an interconnected polyethylene greenhouse with tomato cultivation. The authors performed a feasibility study, which has once again demonstrated the potential of ANNs as an accurate forecasting tool. The inputs for the three neural network models were chosen as the outside temperature, relative humidity, solar radiation and wind speed. In the first two neural models analyzed in the paper, only one output is considered, *i.e.*, temperature or relative humidity. The last model takes into account both variables as outputs at the same time. Various aspects described in the aforementioned papers may be considered in the proposed study.

The present work introduces a new method for calibrating neonatal incubators based on multilayer perceptron (MLP) neural network models, with a reduced number of sensors if compared with that used in the traditional calibration procedure. The basic idea is the use of an ANN to infer the temperature and humidity of the extra sensors positioned inside the equipment according to standard IEC 60601-2-19 by using only the sensors included in the commercial neonatal incubators. The proposed method has four significant advantages over the standard calibration process: (i) while the standard technique requires five temperature sensors and one humidity sensor, the proposed method dramatically reduces the number of sensors to one for temperature and one for humidity; (ii) the calibration process works with the incubator under operation; (iii) no hardware calibration is necessary, and the sensors used in the new process are already installed in the traditional incubator; and (iv) the necessity for calibration is diagnosed in real time, and the presence of technical experts in the NICU is not necessary.

This paper is organized as follows. The next section presents a brief description of the incubator used in this research and the traditional calibration procedure for such equipment. In Section 3, a summary of the main points established by the IEC standard is presented. The theoretical referential of MLP and inferential systems is presented in Section 4. Finally, the simulations and relevant results are discussed in Section 5, while the main conclusions are given in Section 6.

## 2. Theoretical Background of Neonatal Incubator and Calibration System

**Figure 1.**
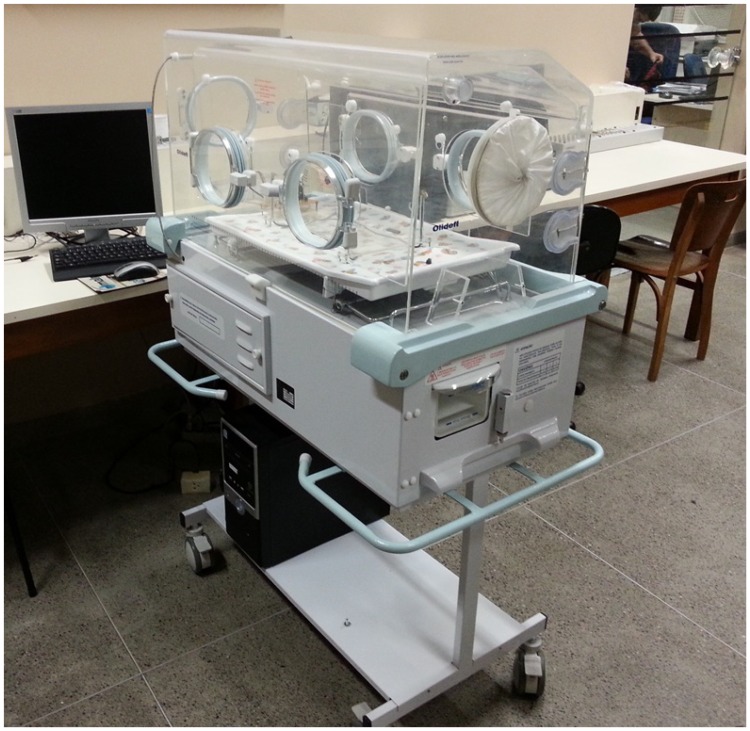
Example of a commercial incubator.

A neonatal incubator, which is represented in Figure 1, consists of a rigid box built in fiber and steel, where an infant may be kept in a controlled environment for medical care. The device includes an AC-powered heater, an electrical motor fan to circulate the warmed air, a water container to add humidity, a mechanical filter through which the oxygen flows and an access port for nursing care. The electric motor allows the air to circulate into the neonatal incubator through an air inlet at the bottom of the equipment. It influences the temperature and humidity levels inside the incubator dome, as well as the oxygen level. The air is renewed by a set composed of an electrical exhaust fan and an air inlet.

**Figure 2.**
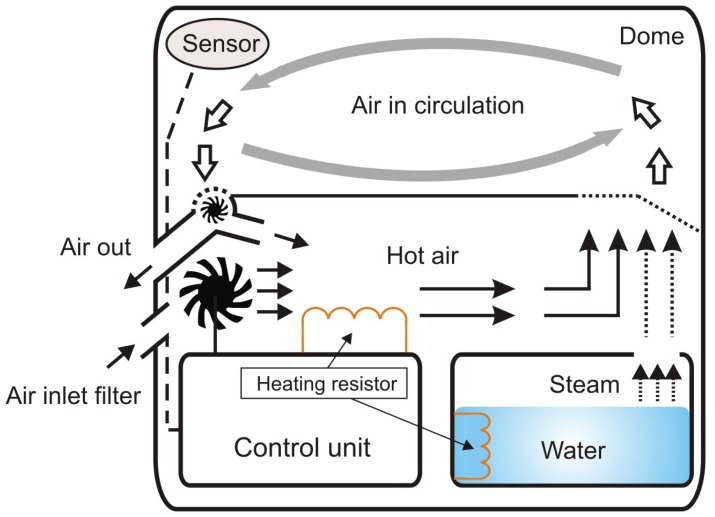
Overview of the incubator operation.

The arrangement is a multivariable system characterized by nonlinearities and interaction among the variables. Within this context, two variables are the most important ones: temperature and humidity, which are regulated by controlling the electric current that flows through two resistors. Temperature and humidity are measured by sensors positioned on the dome. Figure 2 shows a schematic diagram of the overall operation of a neonatal incubator.

**Figure 3.**
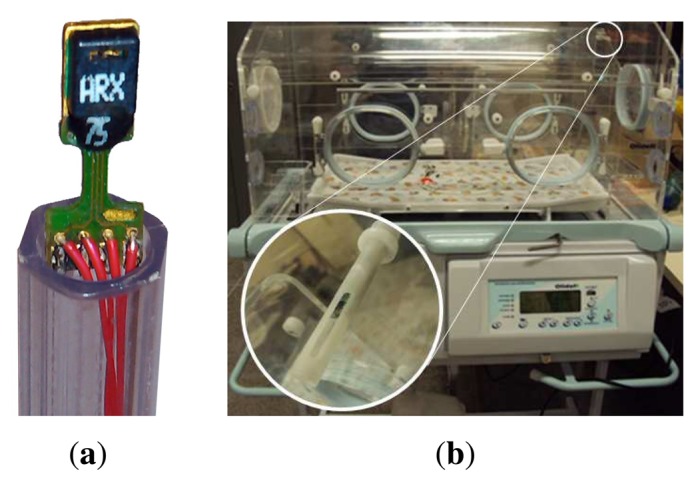
(**a**) Sensor SHT11; (**b**) position of sensor SHT11 in the dome.

The relative humidity sensor, SHT11, represented in Figure 3a, is used, which is placed inside the incubator as shown in Figure 3b. SHT11 integrates a sensor element and a signal processor in a tiny footprint, providing a fully-calibrated output. This sensor is composed of two sensor elements: a capacitive sensor to measure the relative humidity (resolution = 0.05%, accuracy = ±3.0%) and a band-gap sensor to measure the temperature (resolution = 0.01 °C, accuracy = ±0.4 °C). According to the manufacturer (Sensirion), CMOS (complementary metal-oxide-semiconductor) technology associated with an analog-to-digital (A/D) converter and a serial interface circuit provides superior signal quality, a fast response time and insensitivity to external disturbances.

To perform the standardized calibration procedure, an embedded digital system was designed. The system is composed of a data acquisition platform based on the microcontroller PIC18LF2620 and a data communication wireless link based on ZigBee protocols. The embedded system is connected to a PC (personal computer) through a ZigBee radio link. This embedded system is responsible for monitoring the sensor states and sending commands to the power drivers that control temperature, humidity and oxygen levels and the air flowing inside the neonatal incubator. The main objective of the embedded system is to provide the data acquisition and the communication link with the PC.

**Figure 4.**
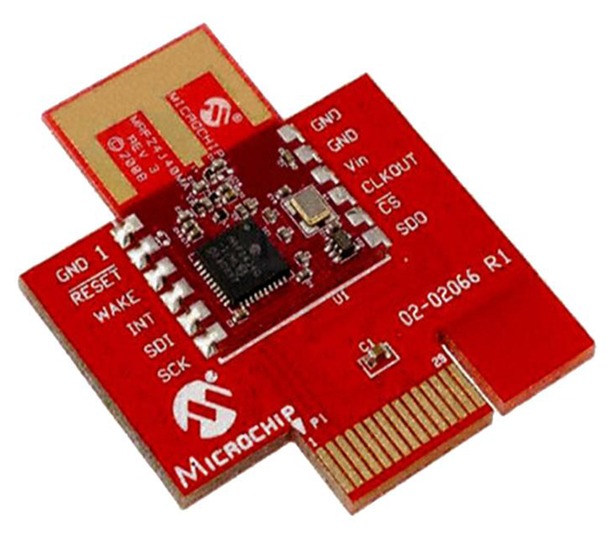
Zigbee radio MRF24J40MA.

The communication link is a ZigBee network, which is a low-cost, low-powered wireless network standard (IEEE–Institute of Electrical and Electronics Engineers-802.15.4). The reduced cost allows the technology to be widely used in wireless control and monitoring applications. The low-powered ZigBee devices often transmit data over long distances by transferring data through an intermediate device in a mesh configuration. ZigBee operates in industrial, science and medical radio bands. The prototype uses the ZigBee radio MRF24J40MA (Microchip), which is shown in Figure 4.

**Figure 5.**
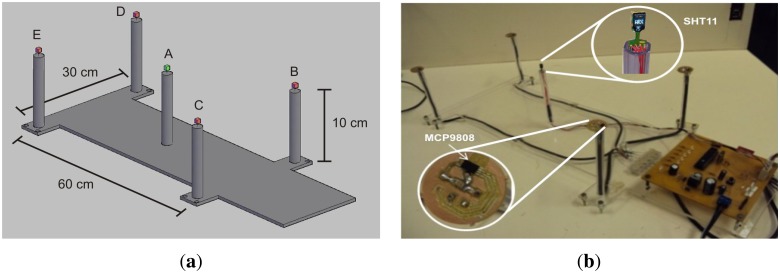
(**a**) Representation of the data acquisition system base; (**b**) experimental setup of the data acquisition system base.

The designed microcontrolled system is based on standard IEC 60601-2-19. This standard specifies the behavior of the temperature in specific points inside the incubator. The aforementioned points are outlined in Figure 5a and named as A, B, C, D and E. Besides temperature, relative humidity must be also measured in central point A.

The mechanical part of the calibration system is comprise of a rigid base made of acrylic, five supports for sensors arranged according to the standard recommendation (given in Section 3) and a control board. At points B, C, D and E, the temperature sensors, MCP9808 (Microchip), are placed. Point A integrates a temperature and humidity sensor, SHT11. The acrylic base with sensors for calibration purposes must be positioned inside the incubator during the tests and removed from the dome when the incubator is under normal operation. The sensor system that integrates the commercial incubator is maintained during the test. Figure 5b presents the experimental setup for the data acquisition system.

## 3. Overview of Standard IEC 60601-2-19

The parameters for quality and safety in Brazilian neonatal incubators must comply with standard IEC 60601-2-19 “Particular requirements for the safety of neonatal incubator”. This standard determines the variation range of environmental variables in the neonatal incubator, such as temperature, humidity, velocity of air flow, and noise level.

**Figure 6.**
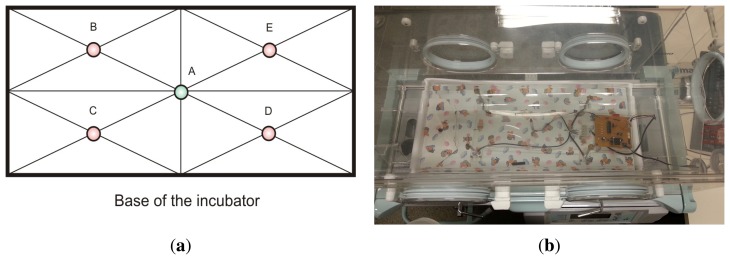
A scheme (**a**) and an overview of the sensor positioning inside the neonatal incubator (**b**) according to standard IEC 60601-2-19.

According to the referred IEC standard, five positions within the incubator are defined to carry out the measurements of temperature and relative humidity, as shown in Figure 6. The standard defines a measurement plane placed 10 cm above of the incubator mattress, where a set of five measurement points (A, B, C, D, and E) exist. Five temperature sensors must be positioned at points A, B, C, D, and E, while the humidity sensor must be positioned at central point A.

ccording to standard IEC 60601-2-19, besides the correct positioning of the sensors, a set of six steps must be followed to calibrate the incubator, which are:

1. Before starting the tests, verify if the environment temperature is between 21 °C and 26 °C.
2. Adjust the incubator temperature control (set by the operator) to 11 °C over the environment temperature. The incubator must reach the temperature control at the time instant specified by the manufacturer with a tolerance of up to 20%. The incubator temperature control is measured by the incubator sensor.
3. Adjust the incubator temperature control to two operation points, *i.e.*, 32 °C and 36 °C. At each point, wait for the stabilization temperature condition (the measured incubator temperature at point A must not vary more than 1 °C within the period of one hour).
4. In step number three, the average temperature measured by the sensors at points A, B, C, D and E cannot differ by more than 0.8 °C from the average temperature of the incubator within the period of one hour. Moreover, the average temperature cannot differ more than ±1.5 °C from the incubator control temperature. The incubator average temperature is obtained by means of temperatures measured in regular intervals.
5. With the incubator temperature at 32 °C, set the temperature control reference in steady state to 36 °C and verify if the overshoot is below 2 °C and that the setting is reached in less than 15 min.
6. The humidity value shown by the incubator sensor must not differ by more than 10% of the value measured by the sensor at position A during the whole period of the incubator functioning.

The aforementioned procedures must be performed periodically after each maintenance procedure or when the incubator sensors are found to be uncalibrated. The accuracy of temperature and humidity measured by the designed embedded digital system (Section 2) are given by the IEC standard and listed in Table 1, whose specifications are based on certified equipment.

## 4. Inferential System Based on MLP

Inferential systems (or soft sensors) represent an attractive approach to estimate the primary process variables [17], particularly when conventional hardware sensors are not available or when high cost or technical limitations complicate on-line use [18]. Inferential estimators make use of easily available process knowledge, including a process model and measurements of secondary process variables, so that primary variables of interest can be estimated in real time [19].

Inferential systems are mathematical algorithms that have been used in a wide range of applications in industry, such as supervision, control and optimization of processes due to their advantages over some physical sensors that present measurement problems. Such systems provide faster measurements with more accuracy and reliability at a lower cost in terms of development, implementation and maintenance.

According to Warne *et al.* [20], despite the wide use of inferential models in practical applications, only a few techniques are discussed in the available literature. Generally speaking, there are mainly three approaches for building inferential models: mechanistic modeling (first principles), statistical regression and artificial intelligence modeling. In this work, the intelligent technique of a multilayer perceptron (MLP) neural network is applied to design the inferential system.

**Figure 7.**
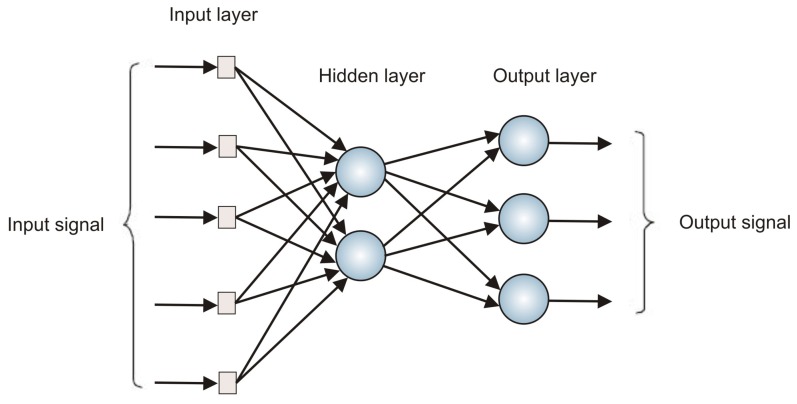
Structure of a multilayer perceptron (MLP).

The MLP neural network architecture consists of a set of neurons arranged in layers, where a layer sends its outputs only to the neurons of the next layer. In addition, all neurons in a given layer are connected to all neurons of the next layer through adjustable parameters of the network, called synaptic weights. These connections are responsible for the MLP's ability to learn and store the knowledge extracted from the problem context. Figure 7 shows the basic structure of an MLP.

An MLP has at least one intermediate layer, called a hidden layer. If only one hidden layer is used, the MLP is able to solve problems of low complexity. Cybenko [13] demonstrated that any continuous function can be approximated by an MLP consisting of a single hidden layer with sigmoid activation functions and an output layer formed by linear neurons. The author concludes that there is no need to use more than one hidden layer and/or a mixture of different types of activation functions. However, the use of more sophisticated architectures may be more appropriate in cases of high complexity mappings.

The synaptic weights between the MLP layers are adjusted through a process called learning or training. The supervised training process can be applied to the MLP architecture to solve mapping problems. In this learning process, the synaptic weights are adjusted based on a set of experimental input-output pairs of the problem and a cost function [12]. A commonly used cost function in the training algorithm is the MSE (mean squared error). In this case, the algorithm tries to minimize the MSE between the neural network output and the target value over all the training examples. Algorithms, such as backpropagation, Levenberg-Marquardt and the Kalman filter, among others, can be used to minimize the cost function in the learning process of this network [21,22].

A supervised neural network requires an external source to provide information about the analyzed problem. This information is given by a set of *N* pairs of vectors, {**x**(*n*), **d**(*n*)}, *n* = 1, 2, …, *N*, where **x**(*n*) ∈ ℝ^(^*^p^*^+1)^ represents the input vector at discrete time *n* and d(*n*) ∈ ℝ*^m^* denotes the desired vector of outputs (responses) for such an input vector.

Each input vector is represented as:

(1) $$\text{x}(n)=\left(\begin{array}{c} x_{0}(n)\\ x_{1}(n)\\ \vdots \\ x_{p}(n) \end{array}\right)=\left(\begin{array}{c} -1\\ x_{1}(n)\\ \vdots \\ x_{p}(n) \end{array}\right)$$

where *p* > 0 denotes the effective number of input variables used in the problem. The term “effective” is used here, because the input component, *x*_0_(*n*), is not really a variable in the usual sense, since it has a fixed value equal to −1 and is known as the threshold or (*bias*). Similarly, the *n* – *th* output vector at a discrete time is represented as follows:

(2) $$\text{d}(n)=\left(\begin{array}{c} d_{1}(n)\\ \vdots \\ d_{m}(n) \end{array}\right)$$

where *m* > 0 indicates the number of output variables in the neural network. Any component of the input vector is symbolized by *x_j_*(*n*) ∈ ℝ, while the component of a given vector output is symbolized as *d_k_*(*n*) ∈ ℝ.

For a given problem, it is considered that vectors x(*n*) and d(*n*) are related according to some unknown mathematical relationship, **f**(·):

(3) d(n)=f[x(n)]

For this purpose, one can use a supervised neural network, such as MLP, by adjusting its synaptic weights during the training procedure to generate a mathematical model that acts as an approximation of **f** (·), denoted by **fˆ** (·):

(4) $$y(n)=\hat{f}[x(n)]$$

where one expects output y(*n*) generated by the neural network to be very close to the desired output d(*n*), even when the inputs presented to the network are different from those used in the training process. This is possible because of the generalization ability of neural networks.

The main goal of this work is to use the supervised neural network MLP to infer the humidity and temperature values at the points specified by IEC 60601-2-19. Thus, in the learning process, vector d(*n*) represents the real behavior of such variables and y(*n*) correspond to the values inferred by the network.

According to IEC 60601-2-19, the humidity and temperature must be measured by a set of sensors positioned inside the neonatal incubator during its calibration. In the traditional procedure, the incubator must be put out of service, which reduces the working time of this equipment. Within this context, it is expected that the inferential systems can be used to evaluate the incubator conditions and the calibration in an optimized way. It is then reasonable to assume that the inferential system can replace the traditional calibration system based on sensors installed inside the incubator.

The proposed neural inferential system adopt the values of humidity and temperature (secondary variables) as inputs, which were instantaneously measured by the sensor, SHT11, available in a commercial incubator. By using measurements and a trained MLP, the system is able to estimate primary variables, which are: humidity at point A and temperature at points A, B, C, D and E, as shown in Figure 6.

**Figure 8.**
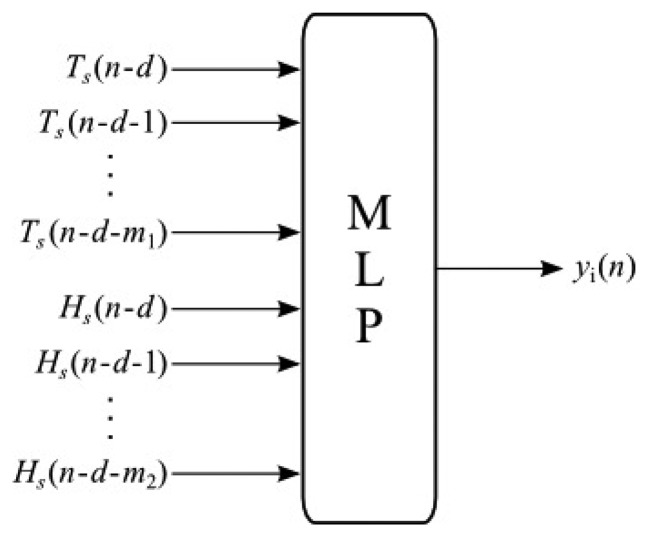
Structure of the MLP inferential system.

The general structure of MLP inferential systems is shown in Figure 8, where *T_s_* and *H_s_* are the measurements of temperature and humidity at the incubator's dome output, respectively, *d* is the transport delay of the system and, finally, *m*1 and *m*2 are the dimensions of the regressors applied to the incubator's secondary variables. At the output of the structure, *ŷ_i_* is the estimation of the i-th primary variable provided by the *i*-th inferential system, according to Table 2.

## 5. Results and Discussion

The results and the methodology used are detailed in the next two subsections. In Section 5.1, the analysis of the results obtained by the neural network is presented. The results are related to the inferential values of the temperature and humidity inside the incubator. In Section 5.2, the procedural steps for the evaluation and calibration of the incubator with the inferential MLP system are presented.

### 5.1. Results Obtained by Neural Network

For the training and testing of the MLP network, 900 samples of temperature and humidity were acquired from points A, B, C, D and E and the incubator sensor, with sampling periods of 24 s. The collected data were divided into two sets: one for training, containing 750 samples and 150 samples for tests. The samples were collected by applying two PRBS (pseudo-random binary sequence) electric current signals to the incubator resistors. The amplitude limits of the PRBS electric currents are set by IEC 60601-2-19 for humidity and temperature inside the incubator. Main text paragraph.

The humidity (*H_S_*) and temperature (*T_S_*) values measured by the incubator sensors at the air outlet of the dome were used as input data in the network, aiming to infer the magnitudes measured by the humidity sensors at point A (*H_A_*) and temperature sensors at points A, B, C, D and E (*T_A_*,*T_B_*,*T_C_*,*T_D_* and *T_E_*, respectively). Therefore, the IEC 60601-2-19 standardized temperature and humidity points can be determined by the inferential MLP process using only the temperature and humidity values (*H_S_* and *T_S_*) measured by the incubator sensor.

**Figure 9.**
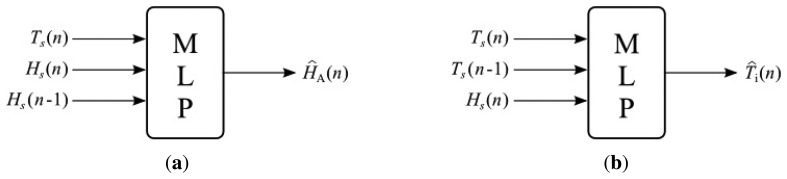
Training structure of networks: (**a**) humidity and (**b**) temperatures.

The values of *H_S_* and *T_S_* are used to assemble the arrays containing the data to be used as network inputs, which must be associated with the desired outputs, *H_A_*,*T_A_*,*T_B_*,*T_C_*,*T_D_* and *T_E_*. The structures for training and validation are presented in Figure 9. Parameters *T_S_*(*n*), *H_S_*(*n*) and *H_S_*(*n* — 1) are used in Figure 9a to estimate humidity *Ĥ_A_*(*n*).Figure 9b uses *H_S_*(*n*), *T_S_*(*n*) and *T_S_*(*n* — 1) to estimate humidity *Tˆ_i_*(*n*)), where *i* is the reference temperature at points A, B, C, D and E. Such values are used in the training process of the network based on a backpropagation algorithm [23]. After the training of all neural networks, the next step consists of evaluating the performance of inferential networks through the MSE of the inferred variables with respect to their desired values. For this purpose, a tool known as boxplot is used to qualitatively evaluate the distribution of errors.

Boxplot (or box diagram) is a graphical way to represent numerical data aligned horizontally or vertically through five quantities: the smallest observation, lower quartile, median, upper quartile and largest observation [24]. The boxplots also show the outliers, which are represented here by the symbol “+”. Outliers are values numerically distant compared to other existing data, which may assume values too small or too large that are located beyond the percentiles.

**Figure 10.**
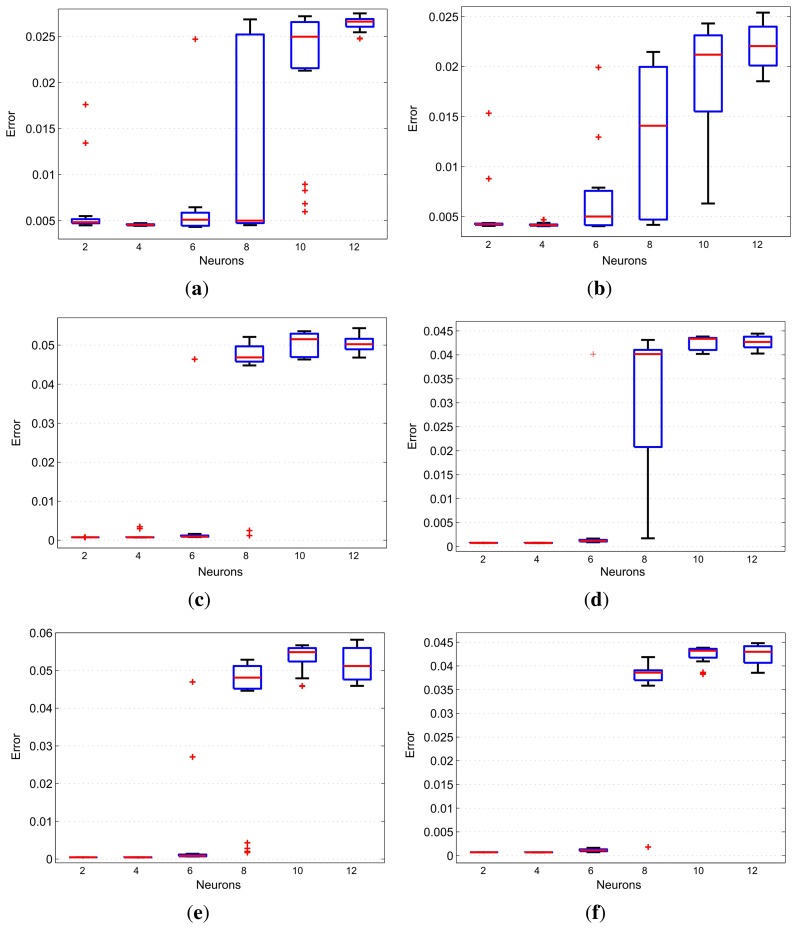
Number of hidden layer neurons: (**a**) humidity at point A; (**b**) temperature at point A; (**c**) temperature at point B; (**d**) temperature at point C; (**e**) temperature at point D; (**f**) temperature at point E.

Figure 10a presents the boxplots for the MSE values of variable humidity in the incubator obtained from a variation in the number of neurons in the hidden layer. These results demonstrate that two or four neurons represent the best model. However, the adoption of four neurons is more interesting, because the model with two neurons has outliers. Figure 10b shows the result for variable *T_a_*. It can be seen that the boxplots for this variable provide results that are very close to those for variable *H_a_*.

**Figure 11.**
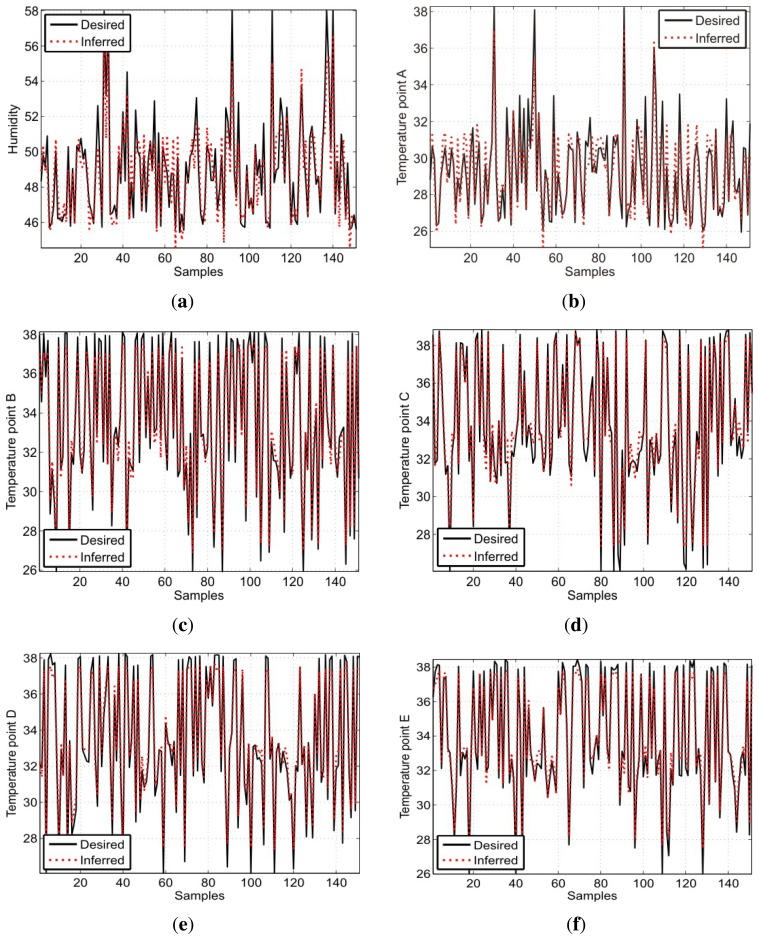
Response of the neural model: (**a**) humidity at point A; (**b**) temperature at point A; (**c**) temperature at point B; (**d**) temperature at point C; (**e**) temperature at point D; (**f**) temperature at point E.

By analyzing the boxplots in Figure 10c–f, for the temperature at points B, C, D and E, one can notice that there are smaller errors for two, four and six neurons. The choice of six neurons can be disregarded by presenting a median slightly larger than the other choices and the presence of extreme values. Thus, in order to infer the temperatures, it can be stated that the choice of four neurons in the hidden layer is the best option to achieve the best models. Therefore, the test results for the aforementioned structures are given in Figure 11a–f.

From the aforementioned results, it is clear that MLP has succeeded in inferring the humidity presented by the sensor at point A and, also, the temperatures given by the sensors positioned at all points specified by the standard. At points B, C, D and E, the network has the best performance, with the MSE ranging between 4.53 × 10^−4^ and 7.69 × 10^−4^. The results for each network are detailed in Table 3, which shows the average MSE and the error variance for both the training and validation of the networks. These values are obtained from the completion of 30 training and validation steps for each variable to be estimated.

The results show higher values of MSE for the temperature and humidity at point A. The temperature MSE at this point is 4.18 × 10^−3^ and the MSE for humidity is 4.55 × 10^−3^. Point A corresponds to the central position of the incubator, as it tends to present higher nonlinearity in the dynamics by being more susceptible to variations. This is less likely to happen at points B, C, D and E, which are located at the ends of the incubator and are less susceptible to temperature variation.

Regarding the analysis of the aforementioned results, the calculation of the relative percentage error between the desired values and the inferred network was performed. In terms of this error, there is the same trend as the MSE, *i.e.*, the humidity and temperature at point A represents a variation of temperature slightly higher if compared to the other specified points. At point A, the moisture presented a relative percentage error ranging from −5% to +5% and a temperature error ranging from −7.5% to +8.0%. At other points, the variation is always within the range of −3.0% to +6.0%, showing that the difference between the desired and inferred temperatures is small. This demonstrates that the MLP achieved satisfactory performance, serving as an effective tool for evaluating the calibration of sensors in the incubator.

### 5.2. Assessment of the Proposed Procedure

**Figure 12.**
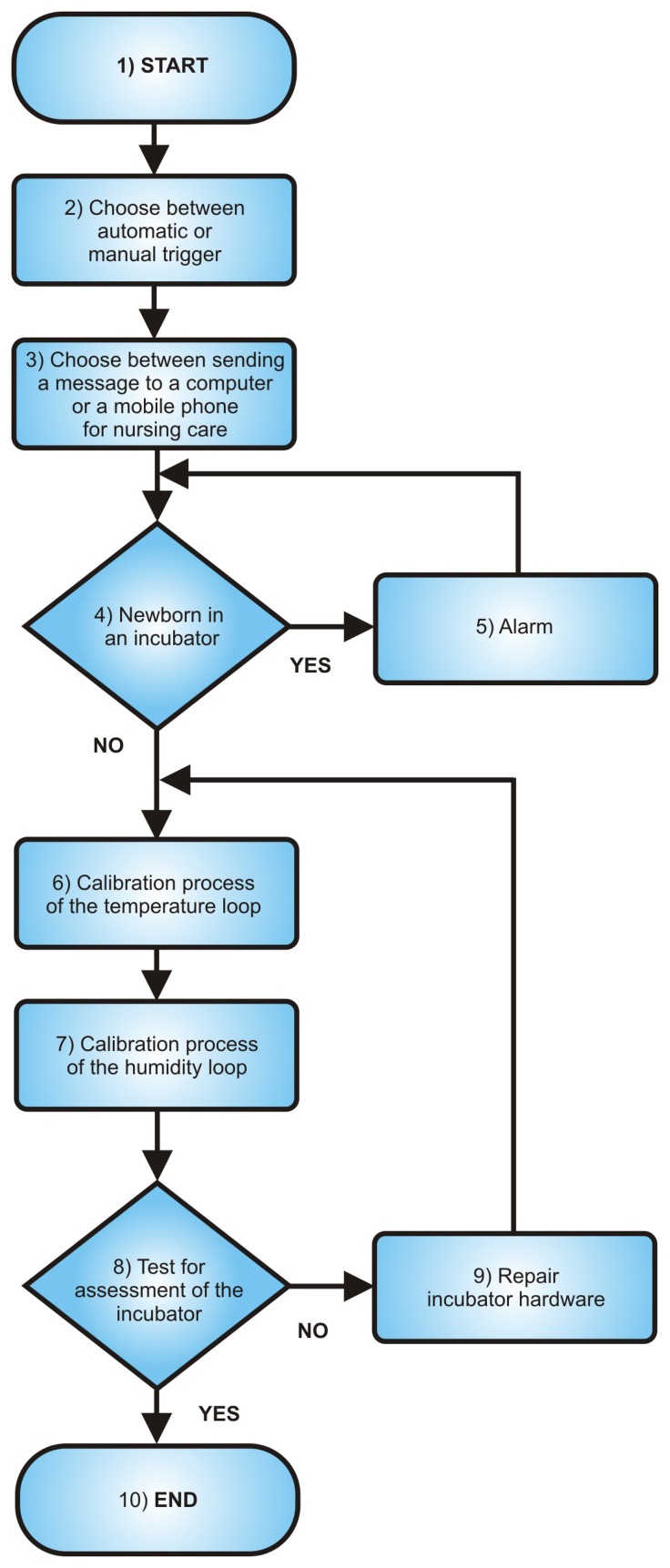
Flowchart representing the calibration process.

The aforementioned inferential neural network system can be used to implement a software-based calibration system as a substitute or a complementary strategy to hardware calibration. The steps described in the previous section can be summarized as the following instructions in the form of the flowchart presented in Figure 12.

When the software detects that the incubator control loops are not within the standard specified range through the MLP inferential sensors, a warning is automatically sent to the NICU computer and to the responsible nurse's mobile phone number, notifying that the incubator needs to be calibrated. This trigger may also occur manually, when the operator detects that the incubator is operating under a nonstandard state. After sending the alert message, the software checks if there is a newborn in the incubator. This check is performed by means of a load sensor. If the presence of a newborn is detected, an alarm signal is triggered, which persists, until the newborn is removed. Then, the software automatically sends a command that starts the calibration procedure of the temperature and humidity loops. Finally, a test is performed to check whether the temperatures at points A, B, C, D and E and, also, humidity at point A are adequate after the calibration process. Numerically, this test results in Tables 4 and 5. According to the results presented in the aforementioned tables, the proposed system achieved a satisfactory performance, satisfying the requirements of the standard IEC 60601-2-19. In other words, the temperature inferred by the MLP network at points A, B, C, D and E does not differ from the neonatal incubator average temperature by more than 0.8 °C, and this temperature does not differ more than ± 1.5 °C from the incubator control temperature. Compared to the established technique, the estimation error is negligible, as shown in the fourth column of Tables 4 and 5. Regarding the humidity, its variation was below 10% of the reference value, which satisfies the IEC 60601-2-19 standard. According to this standard, the reference value for relative humidity is 48%.

## 6. Conclusions

The proposed neural network inferential calibration process of neonatal incubators can be used as an option other than the traditional calibration system based on hardware sensors. This inferential method based on standard IEC 60601-2-19 has shown the advantages of such a method over the hardware calibration process, such as the fact that only one temperature and one humidity sensor are used, that it is not necessary to turn off the incubator to evaluate the incubator condition, that if a more accurate calibration is necessary, it can be detected in runtime, and that during the occurrence of a process warning, alert signals can be sent directly to NICU personnel for repair. Therefore, the intelligent sensor system based on an inferential neural network can reduce the period of the evaluation and calibration of the incubator.

Regarding the accuracy of the estimated values, the neural network architecture has shown small errors with respect to the actual values for humidity and temperature. Therefore, it can be stated that this technique allows for the accurate identification of the dynamic model of the incubator, which is capable of providing estimation in possible advanced risk cases, such as the temperature being out of a safe range and failures in the heater system and/or air flow in the equipment.

With the use of the incubator over the years, it may be required that an MLP retraining be performed, due to natural changes in the operational conditions of this equipment. This is supposed to be the unique limitation of the proposed system. This limitation could be overcome with the implementation of a more sophisticated self-training MLP algorithm.

## Figures and Tables

**Table 1.** Precision in the measurements of temperature and humidity specified by standard IEC 60601-2-19.

**Requirement**	**Precision Required by the Embedded Digital System**
Temperature measurement. IEC 60601-2-19, Section 8, Item 50.101.2	The measured temperature must not differ from the standard temperature thermometer in more than 0.8 °C. The accuracy of the standard thermometer must be at least 0.05 °C
Measured of humidity inside the incubator. IEC 601-2-19, Section 8, Item 50.110.	The relative humidity must not differ more than 10 % from the value measured by a standard equipment.

**Table 2.** Primary variables of the neonatal incubator.

**Index*i***	**Primary Variable*y****_i_*	**Description**
1	*H_A_*	Humidity at point A
2	*T_A_*	Temperature at point A
3	*T_B_*	Temperature at point B
4	*T_C_*	Temperature at point C
5	*T_D_*	Temperature at point D
6	*T_E_*	Temperature at point E

**Table 3.** Results for the analyzed inference MLP systems.

**Inferred Variables**	**Neurons**	**Training**		**Validation**	
		**Mean**	**Variance**	**Mean**	**Variance**
*H_A_*	2	5.31 ×10^−3^	9.21 ×10^−6^	5.93 ×10^−3^	1.12 × 10^−5^
	4	4.15 ×10^−3^	7.55 ×10^−9^	4.55 ×10^−3^	9.19 × 10^−9^
	6	5.49 ×10^−3^	1.56 ×10^−5^	6.10 ×10^−3^	1.97 × 10^−5^
	8	1.17 ×10^−2^	8.16 ×10^−5^	1.27 ×10^−2^	9.71 × 10^−5^
	10	2.01 ×10^−2^	4.69 ×10^−5^	2.16 ×10^−2^	5.57 × 10^−5^
	12	2.44 ×10^−2^	4.43 ×10^−7^	2.64 ×10^−2^	4.36 × 10^−7^

*T_A_*	2	5.32 ×10^−3^	1.13 ×10^−5^	5.00 ×10^−3^	6.97 × 10^−6^
	4	4.30 ×10^−3^	6.03 ×10^−8^	4.18 ×10^−3^	2.19 × 10^−8^
	6	8.08 ×10^−3^	3.85 ×10^−5^	7.11 ×10^−3^	2.36 × 10^−5^
	8	1.14 ×10^−2^	8.71 ×10^−5^	1.26 ×10^−2^	5.63 × 10^−5^
	10	1.93 ×10^−2^	5.50 ×10^−5^	1.85 ×10^−2^	3.87 × 10^−5^
	12	2.49 ×10^−2^	1.53 ×10^−6^	2.20 ×10^−2^	4.21 × 10^−6^

*T_B_*	2	7.32 ×10^−4^	1.16 ×10^−11^	7.42 ×10^−4^	1.91 × 10^−11^
	4	1.07 ×10^−3^	7.29 ×10^−7^	1.12 ×10^−3^	8.87 × 10^−7^
	6	4.89 ×10^−3^	1.39 ×10^−4^	5.54 ×10^−3^	1.95 × 10^−4^
	8	3.71 ×10^−2^	1.49 ×10^−4^	4.33 ×10^−2^	2.06 × 10^−4^
	10	4.34 ×10^−2^	7.53 ×10^−6^	5.04 ×10^−2^	8.19 × 10^−6^
	12	4.32 ×10^−2^	4.30 ×10^−6^	5.02 ×10^−2^	4.60 × 10^−6^

*T_C_*	2	6.58 ×10^−4^	9.18 ×10^−11^	7.74 ×10^−4^	1.02 × 10^−10^
	4	6.58 ×10^−4^	2.38 ×10^−10^	7.69 ×10^−4^	3.52 × 10^−10^
	6	2.94 ×10^−3^	7.69 ×10^−5^	3.17 ×10^−3^	7.57 × 10^−5^
	8	3.16 ×10^−2^	3.16 ×10^−4^	3.12 ×10^−2^	2.94 × 10^−4^
	10	4.47 ×10^−2^	7.05 ×10^−6^	4.25 ×10^−2^	2.01 × 10^−6^
	12	4.49 ×10^−2^	6.87 ×10^−6^	4.25 ×10^−2^	1.99 × 10^−6^

*T_D_*	2	5.00 ×10^−4^	1.52 ×10^−11^	4.56 ×10^−4^	1.74 × 10^−11^
	4	4.93 ×10^−4^	2.95 ×10^−10^	4.53 ×10^−4^	2.76 × 10^−10^
	6	4.02 ×10^−3^	1.02 ×10^−4^	4.53 ×10^−3^	1.33 × 10^−4^
	8	3.37 ×10^−2^	2.62 ×10^−4^	3.97 ×10^−2^	3.67 × 10^−4^
	10	4.43 ×10^−2^	4.00 ×10^−6^	5.36 ×10^−2^	1.03 × 10^−5^
	12	4.29 ×10^−2^	6.11 ×10^−6^	5.16 ×10^−2^	1.73 × 10^−5^

*T_E_*	2	6.46 ×10^−4^	1.71 ×10^−11^	6.91 ×10^−4^	4.54 × 10^−11^
	4	6.41 ×10^−4^	2.23 ×10^−10^	6.80 ×10^−4^	2.08 × 10^−10^
	6	9.99 ×10^−4^	5.09 ×10^−8^	1.12 ×10^−3^	6.53 × 10^−8^
	8	3.79 ×10^−2^	7.64 ×10^−5^	3.66 ×10^−2^	6.99 × 10^−5^
	10	4.44 ×10^−2^	5.02 ×10^−6^	4.23 ×10^−2^	3.47 × 10^−6^
	12	4.45 ×10^−2^	6.94 ×10^−6^	4.23 ×10^−2^	4.71 × 10^−6^

**Table 4.** Calibration results using the proposed system; control temperature = 32 °.

**Temperature**	**Mean Temperature Measured by MLP Inference**	**Mean Temperature Measured by Calibration Standard System**	**Error**
Point A	32.13	32.13	0.00
Point B	32.25	32.13	−0.12
Point C	32.59	32.13	−0.46
Point D	32.44	32.13	−0.30
Point E	32.46	32.13	−0.33

**Table 5.** Calibration results using the proposed system; control temperature = 36 °.

**Temperature**	**Mean Temperature Measured by MLP Inference**	**Mean Temperature Measured by Calibration Standard System**	**Error**
Point A	36.34	36.34	0.00
Point B	36.56	36.35	−0.21
Point C	36.88	36.36	−0.52
Point D	36.44	36.37	−0.07
Point E	36.67	36.38	−0.29
